# Natural Deterioration Processes of *Salix psammophila* Sand Barriers in Atmospheric Exposure Section

**DOI:** 10.3389/fpls.2022.850391

**Published:** 2022-04-08

**Authors:** Ruidong Wang, Xiaohong Dang, Yong Gao, Xia Yang, Yumei Liang, Chen Zhao, Xiaoting Duan

**Affiliations:** ^1^College of Desert Control Science and Engineering, Inner Mongolia Agricultural University, Hohhot, China; ^2^Inner Mongolia Hangjin Desert Ecological Position Research Station, Ordos, China

**Keywords:** desertification control, soil and water conservation, sand barrier, natural degradation, atmospheric exposure

## Abstract

The atmospheric conditions of desert environments are important for the protection of *Salix psammophila* Sand Barrier, and these conditions can affect and change the structure and performance of the sand barrier, causing them to lose their wind proofing and sand fixing benefits. In this study, we have first examined the key environmental factors that affect the exposure of *S. psammophila* sand barrier. Then, we assessed how key factors in the desert atmospheric environment affect structural aging and performance. The relative crystallinity and chemical composition changes in the sand barrier were measured by X-ray diffraction (XRD), Fourier transform infrared spectroscopy (FTIR), and X-ray photoelectron spectroscopy (XPS), and the main degradation factors and processes were discussed. The results showed that the degradation degree of the exposed *S. psammophila* sand barrier was mainly affected by moisture and ultraviolet radiation. Lignin was the main component and the source of photodegradation and photodiscoloration observed in the sand barrier. However, other polysaccharides, such as cellulose and hemicellulose, were less affected by photodegradation. The stress generated by alternating desorption-absorption was the main cause of the expansion and contraction, deformation, cracking, and warping observed in *S. psammophila* sand barrier. We also found a series of irreversible changes and losses that occurred, which affected the natural material properties of *S. psammophila* sand barrier exposed to atmospheric conditions for several years. Exposure times between 5 and 7 years were the most important turning point in time for determining the deterioration of the *S. psammophila* sand barrier. Our results highlighted the importance of the interactions between atmospheric factors and the exposed atmospheric sections of the *S. psammophila* sand barrier from the perspective of environmental effects. However, the exact mechanisms of the sand barrier deterioration still need further investigation. Nevertheless, our overall findings advanced the current understanding of the environmental effects of *S. psammophila* sand barrier for ecological restoration and desertification reversal, especially in stressful desert environments.

## Introduction

As one of many global environmental problems in the current world, desertification has increasingly attracted the attention of desertification control workers and researchers all over the world (Mahata and Sharma, 2021). In recent years, in the field of desertification control, relevant researchers have carried out various sand hazard prevention and control technologies suitable for special environments for the prevention and control of wind sand hazards, all of which provide reference experience for the wide application of desertification control model to a certain extent (You et al., 2021). *Salix psammophila* (C. Wang et C. Y. Yang) is a pioneer shrub used for wind proofing and sand fixing benefits. It is an environment-friendly, fast growing renewable resource with good wind erosion and sand barrier properties (Mi et al., 2008). Because of its drought-resistant characteristics, it easily grows in harsh environments with strong winds, sand, and little rain. This species is widely distributed in mobile dunes in the arid areas of northwest China (Gao et al., 2013). However, in extremely harsh sand environments, the growth of *S. psammophila* is restricted and has difficulty surviving. In the field of desertification control, many scientists and technicians choose fresh *S. psammophila* branches that grow healthily for 3–5 years in the stubble period as materials. In addition, they use fresh *S. psammophila* to create various sand willow configuration patterns on the sand surface, prevent wind erosion or sand burial for desertification control, reduce wind damage, and change the wind erosion conditions (Gao and Yu, 1996; Gao et al., 2013). Numerous experiments have also shown that creating finer sand barrier on mobile dunes can advance the soil environment of sand dunes and promote the restoration and growth of vegetation on dunes (Ren et al., 2007; Wang et al., 2008; Zhao et al., 2008).

*Salix psammophila* is a desert plant that can be made into sand barrier. However, they have some drawbacks. For example, because they degrade under special natural conditions and participate in the carbon cycle of natural material resources, when fresh *S. psammophila* are cut, the supply of plant nutrients is removed and the cell tissue dies (Gao et al., 2013). After fabrication into sand barrier, *S. psammophila* is exposed to field atmospheric sand environments for long periods of time. Owing to compounding atmospheric environmental factors such as ultraviolet (UV) radiation, humidity and temperature changes, wind erosion, rain erosion, changes in intermolecular forces and fractures in the molecular chains will occur, resulting in the loss of functional groups, which ultimately destroys the protection performance of *S. psammophila* sand barrier. The protective properties of wood materials are not only closely related to the basic physical and mechanical properties, but also the change of its chemical composition and structure will eventually accelerate the irreversible degradation process of sand barrier (Gong, 2012). Thus, manifestations such as cracking, warping, color darkening, and reduced physical and mechanical properties will have adverse effects on the service life and durability of *S. psammophila* sand barrier (Wang et al., 2021). The aging of *S. psammophila* sand barrier also irreversibly changes their physical or chemical structures as natural biomaterials change their color over time. The color of a natural biomaterial depends on its basic chemical composition, and discolorations are indications of altered chemical components (Hon et al., 1985). In wild sand environments, with increased time and exposure to the air, the aging of *S. psammophila* sand barrier increases and the deterioration of the *S. psammophila* sand barrier intensifies (Wang et al., 2019).

In our previous studies, it was shown that the structure and the performance of the *S. psammophila* sand barrier varied in the different vertical sections of the sand barrier. At the same time, the degradation degree of *S. psammophila* sand barrier in the stable sand-buried section is greater than that in the atmosphere-exposed section and the atmosphere-sand dynamic interface. The serious decay of stable sand that buried a section of *S. psammophila* sand barrier was mainly caused by microbial and fungal activity in the sandy environment. It is speculated that the degradation of the exposed atmosphere may be caused by photoaging in the atmosphere (Wang et al., 2021). Meanwhile, what remains unclear is how the atmospheric environment affects these *S. psammophila* sand barrier and how we can regulate their deterioration by potentially changing their structure and performance. Consequently, this knowledge gap greatly limits our understanding of the deterioration process of *S. psammophila* sand barrier that are exposed to atmospheric desert environments. However, these have not been extensively explored in harsh desert atmospheres environments.

In order to verify this conjecture, the same population in our research group conducted further experiments to confirm the conjecture of the previous research while conducting long-term continuous observation on the basis of the previous research. In this study, we assessed an exposed section of an *S. psammophila* sand barrier and determined its macrostructure, physical and mechanical properties, and main chemical components over time. We also hypothesized that UV exposure, temperature, and humidity changes in desert environments are the main factors that drive the deterioration of *S. psammophila* sand barrier, and that understanding their mechanisms is the key to preventing their degradation. To test these hypotheses, we studied the biological aging of *S. psammophila* sand barrier in environmental conditions in sand field areas. We also detected the chemical groups and elemental changes to the *S. psammophila* sand barrier using X-ray diffraction (XRD), Fourier transform infrared spectroscopy (FTIR), and X-ray photoelectron spectroscopy (XPS) to determine their degradation processes and causes and to find out the key degradation inflection point of atmospheric exposure of *S. psammophila* sand barrier. If this result can be verified, it will further the scientific frontier in the field of desertification control, which can not only evaluate the use of the node of *S. psammophila* sand barrier in desert areas with strong UV radiation but can also determine how we can improve the rational utilization of *S. psammophila* sand barrier resources.

## Materials and Methods

### Study Site

Experimental materials were collected through field research in November 2020 in Hangjin Banner, Ordos, Inner Mongolia. According to years of solar radiation data from NASA satellites, the total annual radiation in this area is 1,675 k–1,706 k Wh/m^2^, and the annual sunshine is 3,161–3,169 h. There is a marked difference in temperature between day and night, with maximum temperatures reaching 30°C, while the average annual temperature is 6.3°C, and the annual average temperature ranges between 5.5 and 9.1°C. The maximum extreme temperature is 38.1°C, and the minimum extreme temperature is −30.5°C, with a frost-free period of 135 days. The elevation is 1,100–1,300 m, and the region is an arid grassland, consisting of a typical continental monsoon climate (Wang et al., 2019). The annual average precipitation is 186–245 mm, and the precipitation is mainly concentrated in the middle of July to September each year, accounting for 60% of the total annual precipitation with annual average evaporation of 2,720 mm. These conditions are typically accompanied by sandstorms with high and frequent wind speeds, especially in the spring. The wind direction is typically northwesterly, with an average annual wind speed of 3.5 m/s and a maximum gale speed of 28.7 m/s. The region also experiences an annual average of 11–25 days with gale winds (Wang et al., 2019). The soil consists of aeolian sand and chestnut and loess soils.

### Materials

**FIGURE 1:**
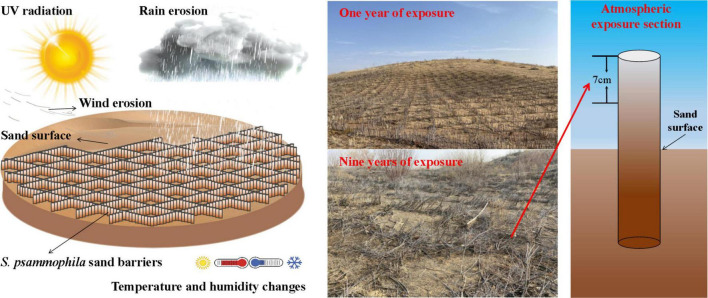
Schematic diagram of the *Salix psammophila* sand barrier sample in atmosphere exposed section.

After knowing the overall condition of the local *S. psammophila* sand barrier, we divided the conditions according to the year they were placed. Sampling was carried out using the method of “space instead of time.” From 2011 to 2020, an *S. psammophila* sand barrier was set up, and samples with 1–9 years of exposure were selected from the samples of *S. psammophila* sand barrier in the atmospheric exposure environment from the sampling site (20 for each iteration). The selected diameter class of *S. psammophila* sand barrier material samples was between 7 < d < 13 mm. These were then collected, and a new *S. psammophila* sand barrier was placed on the gentle sand dune as a control. The schematic diagram of the exposed atmosphere section of the *S. psammophila* sand barrier is shown in Figure 1.

We placed the plant sample of *S. psammophila* sand barrier in the exposed atmosphere section with an intercept of 70 ± 0.2 mm in the climate cabinet. The temperature is set at 20 ± 2°C and the relative humidity is set at 65 ± 5%, according to the requirements of “Test Method for Physical and Mechanical Properties of Sand Shrubs” (Ly/T 2369, 2014), which is the standard of the national forestry industry standard test method. The purpose is to adjust the moisture content to 9-15% and to test the physical and mechanical indexes of the *S. psammophila* sand barrier according to the requirements of the national mechanical test for physical and mechanical properties. In addition, we selected a part of the *S. psammophila* sand barrier samples, removed dust and impurities on the surface of the sand barrier, and ground them into powder. After 40-60 mesh screening, the samples were used to identify the chemical composition, to identify the crystal structure, to identify the spectrum, and for qualitative chemical group analysis.

### Methods

#### Testing of Physical and Mechanical Properties of the *Salix psammophila* Sand Barriers

The indexes of the physical and mechanical properties of the sand barrier were determined by referring to the national forestry industry standard (Ly/T 2369, 2014). We placed the *S. psammophila* sand barrier samples in different exposure times at (103+2)°C for 8 h and then selected 2 ∼3 samples for the first test. The plant sample is considered to be dry when the difference between the last two times of weighing is not more than 0.5% of the sample. When the weight is constant, we use an electronic vernier caliper to measure the length and the diameter of the *S. psammophila* sand barrier samples. We then used an electronic balance with an accuracy of 0.001 g to record their mass and then to calculate the physical characteristics of the *S. psammophila* sand barrier samples. The basic density (g/cm^–3^) and mass loss percentage (%) were estimated from air-dried and oven-dried samples. The mechanical properties of *S. psammophila* sand barrier, such as the modulus of rupture (MOR, MPa) and modulus of elasticity (MOE, MPa), are achieved using universal testing machines (model TY-8000, Tianyuan, Jiangsu, China).

#### Observation of Macrostructure and Microstructure Characteristics

The macroscopic structures of *S. psammophila* sand barrier samples at different exposure times were observed by a Leica Microsystems microscope (Leica SAPO, Leica, Switzerland). We selected the parts with serious degradation and aging in different exposure periods to make structural observation samples and made transverse and radial sections of 5 mm × 5 mm × 5 mm according to the national standard (Gb/T 29894, 2013).

#### Analysis of Main Chemical Components

We selected a part of the crushed *S. psammophila* sand barrier samples to be dried in a drying oven at 80°C after passing through a 40-60 mesh sieve in order to more accurately weigh the constant weight of the samples. Cellulose content was determined by a nitric acid-ethanol method, hemicellulose was adjourned application a hydrochloric acid-DNS abbreviation amoroso method, and lignin was bent application a concentrated sulfuric acerbic band-aid adjustment at an absorption of 72% (Xiong et al., 2005; Wang and Cheng, 2011; Zhang et al., 2016; Yang et al., 2021).

#### Determination of Fourier Transform Infrared spectroscopy

The procedure for making the crushed *S. psammophila* sand barrier sample is described in section “Analysis of Main Chemical Components.” A total of 2 mg *S. psammophila* sand barrier powder samples and 200 mg potassium bromide samples in the atmosphere environment were evenly mixed and placed in a circular mold. We then waited for it to settle for 2 min at 8 to 10 MPa. There were a total of 32 scans of the *S. psammophila* sand barrier samples with an absorbance range of 4,000 to 400 cm^–1^ and a resolution of 4 cm^–1^ (TENSON 27; Bruker Corp., Beijing, China) (Traoré et al., 2016; Boukir et al., 2019; Rudakiya and Gupte, 2019).

#### Analysis of X-Ray Diffraction and X-Ray Photoelectron Spectroscopy

An X-ray diffraction analysis of *S. psammophila* sand barrier samples was carried out by an X-ray diffractometer (Philips X-Pert, Panalytical, Almelo, Netherlands). Then, XPS analysis was performed (Escalab 250Xi; Thermo Fisher Scientific, Waltham, MA, United States). In this study, *S. psammophila* sand barrier with different aging times (0, 5, and 7 years) were selected, and small samples with dimensions of 10 mm × 10 mm × 2 mm (length × width × thickness) were cut. The data were processed using MDI Jade 6.0 and Origin 2021 software. The Cri (%) crystallinity index was described by the Segal method (Segal et al., 1959) using the following equation (Boukir et al., 2019),


(1) $$C\text{r}.I\left(\%\right)=\left[\left(I_{002}-I_{\text{am}}\right)/{\text{I}}_{002}\right]\times 100$$

where *I*_002_ is the diffraction peak intensity of the crystallization corresponding to the (002) plane at 2θ = 22.5°, and *I*_*am*_ is the diffraction intensity at 2θ = 18°.

## Results

### Macroscopic Degradation of *Salix psammophila* Sand Barrier

**FIGURE 2:**
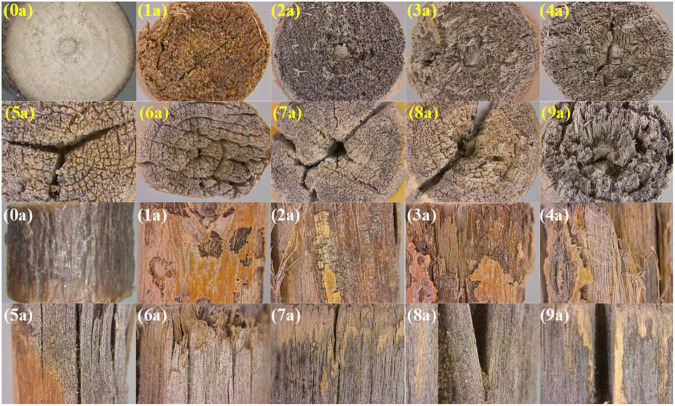
Changes in the macroscopic characteristics of *Salix psammophila* sand barrier (yellow text represents cross sections, and white text represents longitudinal sections).

The macroscopic characteristic changes of *S. psammophila* sand barrier exposed to atmospheric conditions are shown in Figure 2. With increased years of exposure, the *S. psammophila* sand barrier showed different degradation phenomena. The macroscopic observation results showed that *S. psammophila* sand barrier who were exposed for 1 and 9 years experienced the change of different atmospheric environmental conditions (e.g., atmospheric humidity and temperature changes and radiation) and the degradation was considerable. The skin of the unexposed *S. psammophila* sand barrier (0 years) appeared reddish-brown, smooth, without cracks, and had a straight texture. The plant tissue structure of the *S. psammophila* sand barrier was very uniform and integrous, with obvious tree-ring boundaries and uniform distribution of conduits. However, after placement and exposure for 1 year, mild deterioration of *the* sand barrier occurred. The phloem was exposed, and the epidermis had slight cracks and began to fall off. Thus, the results showed that the surface of *S. psammophila* sand barrier changed under the environmental factors after exposure for 1 year, which eventually caused the discoloration of the *S. psammophila* sand barrier. However, the damage was not obvious. The deterioration of the sand barrier gradually increased with increased exposure time, accompanied by different degrees of cracking. After 3–5 years of exposure, the *S. psammophila* sand barrier began to show damages and cracks, and the deterioration became more pronounced. Local microcracks were produced in all of the samples. The cracks all developed from the core of the pith along the radial direction, and the crack type was radial. The color became grayish-brown, and discoloration was obvious. After a series of chemical changes, the surface was damaged and fell off. The outer skin of the *S. psammophila* sand barrier that was exposed for 5 years was completely peeled off and the xylem was seriously cracked. With increased aging time, the number of cracks increased along with the lengths of the cracks. After 7 years of exposure, the tissue of the *S. psammophila* sand barrier was seriously damaged, and its appearance significantly changed. The phloem was completely decomposed, the xylem was completely cracked, and cracks appeared in the form of ring cracks. The lines extended to the adjacent side, and fractures occurred with multiple perforating cracks. Thus, it was obvious from the macroscopic visual observations that the environmental conditions significantly affected the *S. psammophila* sand barrier.

### Physical and Mechanical Properties Analysis of *Salix psammophila* Sand Barrier

**FIGURE 3:**
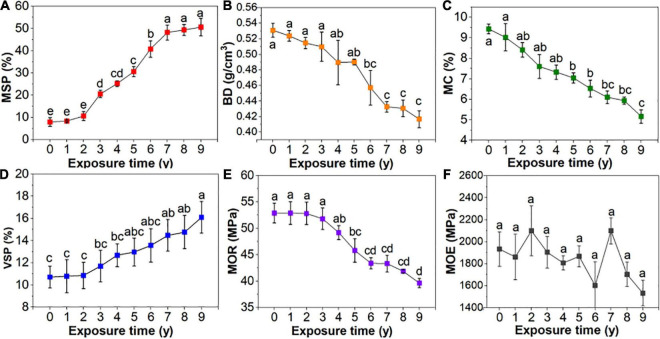
Physical-mechanical properties of *Salix psammophila* sand barrier. **(A)**: Mass loss percentage, **(B)**: Basic density, **(C)**: Moisture content, **(D)**: Volumetric shrinkage percentage, **(E)**: Modulus of rupture, **(F)**: Modulus of elasticity.

Figure 3 shows the relationship between the physical and mechanical properties of the *S. psammophila* sand barrier after years of exposure. With increased exposure time, the mass loss percentage increased, while the physical properties, such as density, moisture content, and volume shrinkage percentage, decreased. The decrease initially occurred quickly but then slowed. According to the various analyses, there were significant differences in the changes of the physical-mechanical properties of the exposed atmospheric section of the *S. psammophila* sand barrier with that of exposure years. In addition, there was a certain correlated relationship (*p* < 0.05). The percentage of increase or decrease for 1–5 years of exposure was faster, and the mass loss percentage and volume shrinkage percentage increased by 22.13 and 20.12%, respectively. The density and moisture content decreased by 6.39 and 21.93%, respectively. After 5–7 years of exposure, the changes slowed down. After 7 years of exposure, the mass loss percentage and the volume shrinkage percentage increased by 48.19 and 34.03%, respectively, while the density percentage and moisture content decreased by 17.37 and 32.37%, respectively. The MOR of the material mechanics performance is the most important resource utilization and, in our study, changes in the mechanical properties, mainly MOR, were consistent with that of the physical properties, which decreased by 13.41% after 5 years and 18.08% after 7 years. Thus, the change rule of the MOR was poor, and overall deterioration exhibited a downward trend after 1–9 years of exposure time. This indicates that exposure time of 5 and 7 years is the most turning point that determines the deterioration of *S. psammophila* sand barrier in atmospheric exposure section.

### Quantitative Analysis of Main Chemical Components

**FIGURE 4:**
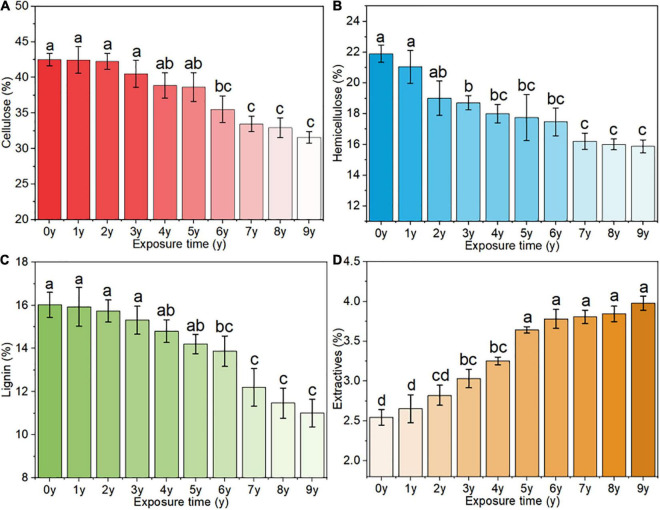
Changes in the main chemical components of the *S. psammophila* sand barrier, **(A)** cellulose, **(B)** hemicellulose, **(C)** lignin, and **(D)** extracts.

In addition to small amounts of extract, *S. psammophila* is mainly composed of cellulose, which is the main skeletal material that provides mechanical strength, while lignin and hemicellulose act as fillers and bonding materials. Figure 4 shows the chemical composition analysis results of *S. psammophila* sand barrier after different years of exposure during long-term environmental use in the sand field area. With increased years of exposure, the contents of cellulose, hemicellulose, and lignin decreased. The decrease initially occurred quickly and then slowed down. The extract content showed an increase, and the changes were slightly fast initially and then slowed gradually. According to the variance analysis, different exposure years of *S. psammophila* sand barrier had a significant difference of the content of cellulose, hemicellulose, and lignin and had a certain correlation (*p* < 0.05). Compared with samples exposed for 1 year, after 5 years of exposure, the contents of cellulose, hemicellulose, and lignin decreased by 8.96, 15.67, and 10.87%, respectively. Furthermore, after 7 years of exposure, their contents decreased by 21.19, 22.99, and 23.43% respectively. Lastly, after 5 and 7 years, the extract response increased by 27.17 and 30.29%, respectively.

### Characteristic FTIR Spectra of *Salix psammophila* Sand Barrier

**FIGURE 5:**
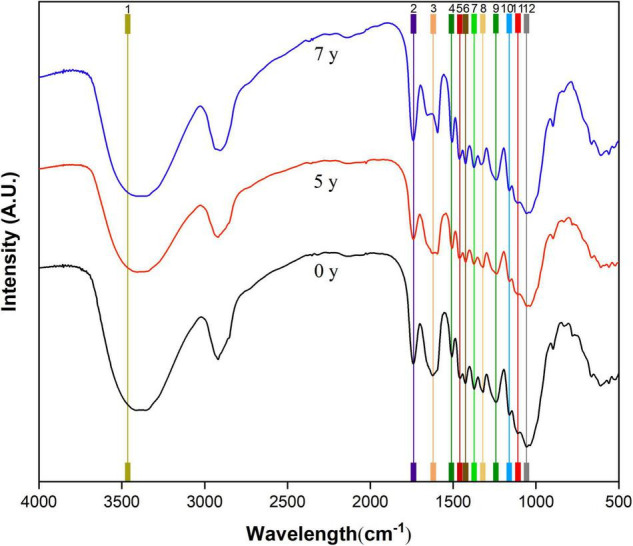
Fourier transform infrared spectroscopy (FTIR) spectra of the *Salix psammophila* Sand Barrier.

To evaluate the changes of the *S. psammophila* sand barrier during natural degradation, infrared spectroscopy was used. The 4,000–400 cm^–1^ functional group regions of the infrared spectrum were the key regions for the analysis and assessment of the various compounds. The infrared spectrum normalized at 3,400 cm^–1^ as shown in Figure 5, and their corresponding components are shown in Table 1. For natural biological materials, chemical changes in the various groups reflect the fingerprint infrared spectrum region (800–1,800 cm^–1^). At 1,739, 1,620, 1,510, 1,460, and 1,425 cm^–1^, there were obvious absorption peaks for the *S. psammophila* sand barrier exposed for 0 years (unaged). The absorption peaks of sand barrier at 5 and 7 years of exposure indicate that vibration occurs with the increase of exposure time. It consists of C = O vibrations of the acetyl group and the carboxyl group of the hemicellulose at the absorption peaks of 1,739 cm^–1^ (Alice et al., 2013), while 1,510 cm^–1^ was correlated to framework vibrations of the aromatic benzene ring of lignin and the stretching vibrations of C = O near 1,620 cm^–1^. The *S. psammophila* sand barrier exposed for 5 and 7 years showed multiple absorption peak intensity fluctuations. However, the characteristic peaks near 1,620 cm^–1^ significantly expanded, which suggests that the carboxyl group of the *S. psammophila* sand barrier of hemicellulose decomposition under the action of enzyme affects the characteristic peaks near 1,242, 1,161, and 1,109cm^–1^ and with relatively small fluctuations (Takei et al., 1997). The *S. psammophila* sand barrier contents of cellulose, hemicellulose, and lignin were degraded with increased years of exposure time.

**TABLE 1:** Fourier transform infrared spectroscopy (FTIR) absorption spectra obtained of the *Salix psammophila* sand barrier and their corresponding components.

Peak no.	Wavelength assignment	Untreated
1	O_2_-H_2_…O_6_ intramolecular stretching in cellulose	3415.78
2	Unconjugated C = O in organic molecules (hemicellulose)	1739.72
3	C = O stretching in conjugated double bonds	1620.13
4	Aromatic skeletal ring in lignin	1510.20
5	C-H deformation in lignin and carbohydrates	1460.05
6	C-H deformation in organic molecules	1425.33
7	C-H deformation in cellulose and hemicellulose	1373.26
8	C_1_-O vibrations in syringyl units and C-H vibrations in cellulose	1321.18
9	C-O stretch in lignin and C-O linkages in guaiacyl aromatic groups	1242.10
10	C-O-C vibrations in cellulose and hemicellulose	1161.10
11	Aromatic skeletal and C-O stretch in lignin	1109.02
12	C-O stretch in cellulose and hemicellulose	1056.95

### X-Ray Diffraction Analysis of *Salix psammophila* Sand Barrier

**FIGURE 6:**
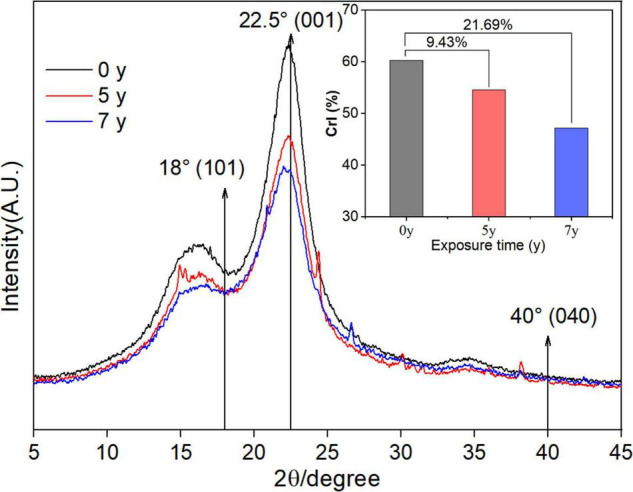
XRD diffractogram of *Salix psammophila* sand barrier.

Figure 6 shows the *S. psammophila* sand barrier exposed for 3, 5, and 7 years of the XRD diagram and relative crystallinity. 18°, 22.5°, and 35° of angle X-ray diffractions (2θ) near the crystal diffraction peak represent the *S. psammophila* sand barrier of cellulose (101), (002), and (040), respectively, on the surface of the crystal diffraction intensity. Among these, the 2θ of 22.5° reflected the crystal diffraction peak of the (002) cellulose plane. Furthermore, with increased exposure time, the angle 2θ that corresponded to the diffraction peak was unchanged and the position remained the same, indicating that, during the deterioration process of the *S. psammophila* sand barrier, the cellulose crystal structure was not damaged. With increased years of exposure, the absorption intensity of the crystal diffraction peak of the *S. psammophila* sand barrier exposure time for 5 and 7 years was lower than the barrier that was unexposed (0 years). The cellulose crystalline content of S. psammophila sand barrier relative crystallization is clearly decreased, and the decreased percentages after 5 and 7 years of exposure were 9.43 and 21.69%, respectively, as shown in Figure 6. The results also indicated that, with the degradation of lignin, some of the cellulose crystallinity in the crystallinity zone was modified into an amorphous state due to the long-term influence of sunlight and environmental humidity, which led to a decrease in relative crystallinity.

### XPS of *Salix psammophila* Sand Barrier

**FIGURE 7:**
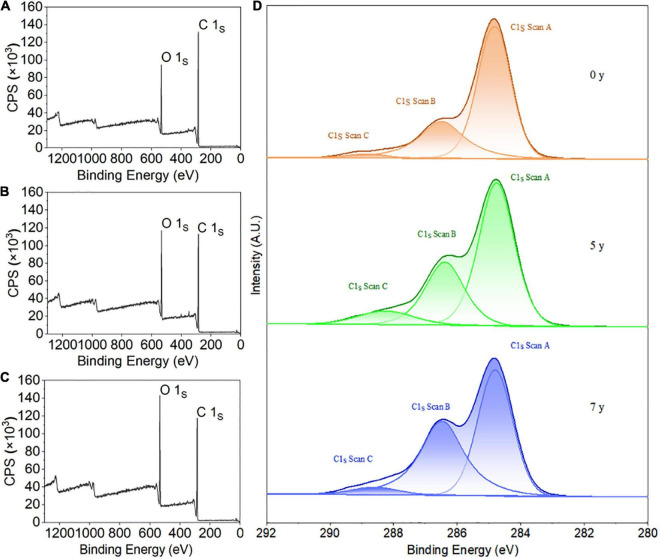
X-ray photoelectron spectroscopy (XPS) survey spectra and C1s narrow scan of *S. psammophila* sand barrier; **(A)** 0 years of exposure; **(B)** 5 years of exposure; **(C)** 7 years of exposure; and **(D)** C1s narrow scan of sand barrier.

X-ray photoelectron spectroscopy characterization of element composition and chemical valence state the characteristics of the common analysis methods, and it can reveal the degradation mechanisms of biomaterials. The main components of natural biomaterials are carbon (C), hydrogen (H), and oxygen (O), along with cellulose, hemicellulose, lignin, and extracts. Figure 7 details the XPS wide sweep spectrum and the peak dividing characteristic curves of C1s of *S. psammophila* sand barrier with increased exposure time. The relative compositions of the various elements are shown in Table 2. As observed in Figure 7, the elemental composition of the sand barrier mainly consisted of C and O elements whose electron binding energies were 285 and 532eV, respectively.

**TABLE 2:** Elemental composition of *S. psammophila* sand barrier (%).

	O	C	C_1_	C_2_	C_3_	O/C	C_1_/C	C_2_/C	C_3_/C
0y	21.54	78.45	51.87	24.63	1.95	27.46	66.12	31.40	2.49
5y	27.35	72.65	47.91	19.12	5.62	37.65	65.95	26.32	7.74
7y	30.07	69.93	36.58	30.54	2.81	43.00	52.31	43.67	4.02

To analyze the chemical bonding states of the sand barrier with different exposure years, the C1s peaks of the high-resolution spectra for the sand barrier exposure time for 0, 5, and 7 years were fitted and divided into peaks and four peaks were obtained (as shown in Figure 7D). This indicated that there were four types of carbon binding states of *S. psammophila* sand barrier, namely, C_1_, C_2_, C_3_, and C_4_. In our study, the content of C_4_ was relatively low, and the fitting analysis of this C atom type was not conducted. The ratio values of the C_1_, C_2_, and C_3_ peak areas of the *S. psammophila* sand barrier are shown in Table 2, and C_1_ and C_2_ were the main binding forms of the C atoms. Specifically, C_1_ is derived from lignin, fatty acids, fats, and wax extracts, whereas C_2_ is derived from the hydroxyl groups (−OH) of cellulose and hemicellulose, and C_3_ is derived from the lipid and carboxyl groups (−C = O) of hemicellulose and lignin. However, changes in C_1_ and C_2_ contents indicated that lignin was more stable than the polysaccharides (cellulose and hemicellulose). Among these, the C_3_ carbonyl group was hydrophilic. With the decrease of *S. psammophila* sand barrier carbonyl content, its main physical-mechanical properties may have changed. Furthermore, the main chemical composition of the *S. psammophila* sand barrier also changed in the atmospheric exposure environment.

As detailed in Table 2, after 5 and 7 years of exposure, the obvious changes of the *S. psammophila* sand barrier have taken place in the relative elements. The content of C decreased, and the content of O increased. Compared with the unaged 0-year exposure sample, the amount of C decreased by 7.98% and O increased by 21.24% after exposure for 5 years. After exposure for 7 years, C content decreased by 12.18% and O content increased by 28.36%. The content of C_1_ obviously decreased from 51.87 to 47.91% after an exposure time of 5 years and to 36.58% after an exposure time of 7 years. The C_2_ content also slightly increased with an increased exposure time, and the content of C_3_ also increased. Furthermore, the degradation of cellulosic materials was analyzed by the changes in the ratios of O to C atoms (O/C), which was quantitatively analyzed when the *S. psammophila* sand barrier was measured by XPS. The oxygen/carbon ratio (O/C) results of the *S. psammophila* sand barrier with different exposure time showed obvious differences. In general, cellulose, which has an approximate molecular formula of C_6_H_10_O_5_, had a high O/C ratio of about 0.83. The O/C value of the hemicellulose, represented by xylan, is about 0.8. The lignin composition of the *S. psammophila* sand barrier is complex, and the theoretical value of O/C is approximately 0.33, while the O/C value of the extract is the lowest at about 0.1 (Barry et al., 1990). The O/C value of the *S. psammophila* sand barrier showed an increasing trend with an extended exposure time, and the value increased from 0.27 to 0.38 after exposure for 5 years and from 0.27 to 0.43 after 7 years of exposure. Based on the analysis of C1 content and the O/C ratio in this study, the content of lignin and extract in the *S. psammophila* sand barrier was lower. In addition, humidity and temperature changes, UV light, and other environmental conditions, along with the inward and outward migration of extracts affected the *S. psammophila* sand barrier’s main chemical composition. Thus, the *S. psammophila* sand barrier dynamic changes have taken place in the surface chemical properties (Wang et al., 2009).

## Discussion

### Changes of Physical and Mechanical Properties of the *Salix psammophila* Sand Barrier

As expected, the results confirm our previous conjecture and support our first hypothesis that the *S. psammophila* sand barrier, as a natural biomaterial, experienced degraded physical and mechanical properties with increased years of exposure. The key time nodes in the process of atmospheric degradation of *S. psammophila* sand barrier were also indicated. Exposure times of 7 and 7 years were the important time points for determining the deterioration of the *S. psammophila* sand barrier. Owing to atmospheric environmental factors, the mass and basic density changes of the *S. psammophila* sand barrier increased its moisture content, and the resulting moisture content of the *S. psammophila* sand barrier increased faster. However, the size of the *S. psammophila* sand barrier decreased with the change of moisture content. The shrinkage phenomenon of natural biological materials will cause the original shape and size of material properties, and the frequent uneven shrinkage will lead to the deterioration of *S. psammophila* sand barrier and other defects, which will further affect the performance of *S. psammophila* sand barrier for wind prevention (Wang et al., 2021). As natural and organic materials, the protection ability of the *S. psammophila* sand barrier and its chemical structure changes are closely related. Owing to external factors, the interaction forces between molecules will change and even fracture long chain structures or functional groups, which eventually leads to the destruction of the aggregated structure and the changes in its physical and mechanical properties (Zhou, 2012). After exposure time for 1–5 years, the physical-mechanical properties of the *S. psammophila* sand barrier rapidly changed. The physical, mechanical, and chemical properties showed that the changes were evident in the first 5 years, and changes were slow after year 5. These results were due to increased water content during this time interval. As the dimensional stability of *S. psammophila* sand barrier changes, its degradation rate is also affected, which leads to the reduction of the protective capability of *S. psammophila* sand barrier. Researchers have shown that natural biological material physical-mechanical performance of decline is caused by moisture and chemical composition of the loss (Ma and Zhao, 2006).

### Main Chemical Composition Changes of the *S. psammophila* Sand Barrier

After exposure for 5 years, the degradation products of the *S. psammophila* sand barrier cellulose and hemicellulose gradually dispersed in the amorphous zone, resulting in an increased weight loss rate and a steady increase in water content. After 7 years of exposure, the main chemical composition and the content of the *S. psammophila* sand barrier degraded to an inflection point, and the products disappeared completely, which reduced the deterioration of the *S. psammophila* sand barrier and slowed the decline of its mechanical properties. The degradation of hemicellulose was the highest, followed by cellulose and lignin. This was due to the structure of hemicellulose, which is composed of short molecular chains and is a type of low molecular weight amorphous polymer polysaccharide that hydrolyzes easily. By contrast, cellulose has a higher molecular weight and crystallinity, and its crystalline regions are closely arranged, making it difficult to degrade. Although lignin is an amorphous polymer, its basic structural unit is styrene-propylene-fired, giving it high chemical stability, and as a result, it is difficult to degrade (Li, 2014). The decrease in the acid-insoluble lignin content of the *S. psammophila* sand barrier indicates that lignin has been degraded, and the most obvious degradation mechanisms of lignin were oxidation and cracking of the aromatic rings.

### Characteristic FTIR Spectra and XRD Diffractogram of the *S. psammophila* Sand Barrier

Fourier transform infrared spectroscopy analysis of the *S. psammophila* sand barrier after exposure for 5 and 7 years was consistent with the observed changes in the chemical groups of lignin under visible and UV light. Due to the esterification reaction of lignin in a special atmospheric environment, the combination of lignin and the carbonyl group and the decomposition of aromatic ring under the action of UV light led to the increase of the carbonyl group (Colom et al., 2003; Li, 2003). Compared to the sample after 5-year exposure, the *S. psammophila* sand barrier infrared spectrum after 7 years of exposure indicated few characteristic peak changes, and the results were similar to the sample exposed for 5 years. The result again indicated that, after atmospheric exposure for 7 years, the chemical composition of *S. psammophila* sand barrier in a stable state. Meanwhile, the infrared spectrum analysis of absorption peak at 1,460 cm^–1^ C-H bond disappearance also indicated lignin photodegradation. The decomposition of the chemical components of the *S. psammophila* sand barrier was promoted by changes to UV light, temperature, and humidity in the field environment over time. In addition, after exposure for 5 and 7 years, the *S. psammophila* sand barrier experienced obvious stretching and vibrations at 1,160 cm^–1^, which was attributed to the stretching vibrations of the C-O-C bond in crystalline cellulose. The results thus showed that the cellulose crystallized and degraded into an amorphous structure during the degradation of hemicellulose and lignin. Previously, Qin Li showed that the photoaging phenomenon of natural biomaterials is due to the chemical composition of natural biomaterials, as they are easily degraded by UV and visible light radiation, resulting in material color changes and degraded mechanical properties (Qin and Yu, 2009). Furthermore, humidity affects natural biological materials due to the moisture absorption-desorption effect, which affects the microstructure and molecular forces in natural biological materials and eventually affects the mechanical properties of natural biological materials and their utility (Yang, 2014; Wang et al., 2021). Furthermore, biological materials occur due to wind and rain erosion (Unger et al., 2001). Similarly, materials degraded by UV light are easily washed away by wind and rain erosion (Neumann, 1998). Natural biomaterials exposed to the atmosphere can also be damaged by erratic fluctuations in humidity and temperature (Borgin et al., 1975b). In general, the influence of these combined factors will cause the material surface to begin to decompose from sapwood to heartwood (Unger et al., 2001).

In general, mechanical properties such as MOE and MOR increase with the increase of cellulose crystallinity of natural biomaterials (Venkateswaran, 2010; Kamarudin et al., 2014). With increased exposure time, the diffraction peak intensities of the *S. psammophila* sand barrier decreased. This indicated that, with the degradation of lignin, part of the crystalline area of cellulose transformed into amorphous cellulose due to the long-term effects of light and environmental humidity (water), leading to decreased relative crystallinity. Notably, our results matched with those of Li et al. (2019). In addition, the peak area method was used to calculate the relative crystallinity. After exposure for 5 and 7 years, the relative crystallinity of the samples decreased by 9.43 and 21.69%, respectively. This was due to the cellulose and half fibers in the chemical composition experiencing more serious degradation. Specifically, as hemicellulose degraded the acetyl groups, the acetic acid it produces destroys the chemical structure of cellulose and reduces the crystallinity (Akguel et al., 2007).

### XPS Spectroscopy of *Salix psammophila* Sand Barrier

After exposure for 5 and 7 years, the chemical element content of the *S. psammophila* sand barrier changed. The content of elemental C decreased, while elemental O increased (Table 1). In terms of the binding of C atoms, the content of C_1_ was significantly reduced, the content of C_2_ slightly increased, and the content of C_3_ increased (Figure 7). Thus, as the set O content increased, the oxidation state of C also significantly increased, and the *S. psammophila* sand barrier contained a number of increased hydroxyl, carbonyl, and carboxyl groups that experienced increased oxidation states and light oxidation reactions. Lignin contains an aromatic structure. Thus, it was the least photostable and was oxidized first. Because lignin is mainly composed of guaiacyl, syringyl, and p-hydroxyphenyl structural units, it easily absorbed UV light, generating free radicals and photooxidation reactions and forming carbonyl or carboxyl groups. This leads to the photodegradation and discoloration of the *S. psammophila* sand barrier. A similar result was observed on the photodegradation effects of wood (Temiz et al., 2007). With the increased exposure time of the *S. psammophila* sand barrier, the O/C value increased from 0.27 to 0.43, which also indicated a decrease in the lignin content. In addition, other polysaccharides were relatively stable and were less affected by photodegradation, such as cellulose and hemicellulose (Wang et al., 2009). Furthermore, the increase in O/C values reflected the decrease in relative lignin content due to hemicellulose degradation, which was consistent with previous chemical composition analyses.

Typically, the aging and degradation of *S. psammophila* sand barrier consist of biodegradation, fungal degradation, photodegradation, and chemical degradation. In our previous study, the degradation phenomenon of *S. psammophila* sand barrier at different vertical positions was explained, mainly in the different damage phenomena of *S. psammophila* sand barrier in atmospheric environment section (Wang et al., 2021). On the basis of previous research, this study further clarified the degradation reasons and degradation rules of *S. psammophila* sand barrier only for the above-ground part (atmospheric environment section). Hence, further analysis of chemical elements was added to fully support the results of this study. From a macroscopic structure standpoint, the *S. psammophila* sand barrier that were exposed to the atmosphere did not show any evidence of cellulose-fed biological nests from desert animals or mycelia of fungi. Thus, we inferred that the *S. psammophila* sand barrier were mainly photodegraded. When the natural biological material was exposed to atmospheric conditions, chemical degradation occurred due to UV radiation, causing serious damage (Tolvaj and Faix, 1995; Papp et al., 2005). Kataoka et al. (2007) and Zahri et al. (2007) showed that the light energy of solar radiation is more harmful to natural biomaterials in the wild. Furthermore, due to the action of water, a series of irreversible changes occurred with exposure time. Changes in ambient temperature, rainfall, and evaporation caused changes in water content within the *S. psammophila* sand barrier. When the internal moisture changed unevenly, stress was generated, causing the *S. psammophila* sand barrier to expand and contract, resulting in deformation, cracking, and warping (Liu and Zhao, 2012; Wang et al., 2020). Thus, from research analysis results, we found that the main degradation chemical components were created by light irradiation under natural conditions and changes in moisture, which was due to the hygroscopic absorption process. However, further studies are needed to assess the specific effects of UV light and moisture changes on the degradation process of *S. psammophila* sand barrier exposed to the environment. Therefore, more robust methods, such as UV radiation and cyclic hygroscopy-desorption, and targeted long-term observations should be considered in future studies.

## Conclusion

Our study showed that the main causes of degradation mechanisms that affected the performance of the *S. psammophila* sand barrier were moisture content and UV radiation. Lignin was also found to be the main component of and the source of the photodegradation and photodiscoloration observed in the *S. psammophila* sand barrier. However, other polysaccharides, such as cellulose and hemicellulose, were less affected by photodegradation. The stress generated by cyclic moisture absorption and desorption was also found to be the reason for the expansion and contraction, deformation, cracking, and warping observed in the *S. psammophila* sand barriers.

Furthermore, the physical-mechanical properties of the *S. psammophila* sand barrier exposed to atmospheric conditions degraded to different degrees with exposure time, and the main chemical components decreased. The change curve patterns exhibited an increase and then plateaued. Exposure times of 5 and 7 years were the important turning points for determining the deterioration of the *S. psammophila* sand barrier exposed to atmospheric conditions. After 7 years, the mass loss percentage and volume shrinkage percentage increased by 48.19 and 34.03%, respectively. In addition, the density percentage and the moisture content decreased by 17.37 and 32.37%, respectively, while the MOR indexes decreased by 18.08%. The content of cellulose, hemicellulose, and lignin also decreased by 21.19, 22.99, and 23.43%, respectively. The severe degree of deterioration resulted in decreased crystallinity, and the crystalline area in the cellulose decreased, affecting the mechanical protective capability of the *S. psammophila* sand barrier. Furthermore, characteristic peaks observed at 1,739, 1,620, 1,510, and 1,460 cm^–1^ corresponded to the vibrations and stretches for lignin fiber and hemifibrin. During the aging process, the content of elemental C decreased, the elemental O and the O/C ratio increased, the oxidation state of C increased significantly, and the content of C and carboxyl groups increased significantly. Therefore, the results of this study may improve our understanding of the degradation processes and causes of *S. psammophila* sand barrier degradation in stressful desert environments.

## Data Availability Statement

The original contributions presented in the study are included in the article/supplementary material, further inquiries can be directed to the corresponding author/s.

## Author Contributions

RW: data curation and writing—original draft preparation. XD: supervision, investigation, and reviewing and editing. YG: conceptualization and validation. XY: software and methodology. YL: date curation. CZ: reviewing and editing. All authors contributed to the article and approved the submitted version.

## Conflict of Interest

The authors declare that the research was conducted in the absence of any commercial or financial relationships that could be construed as a potential conflict of interest.

## Publisher’s Note

All claims expressed in this article are solely those of the authors and do not necessarily represent those of their affiliated organizations, or those of the publisher, the editors and the reviewers. Any product that may be evaluated in this article, or claim that may be made by its manufacturer, is not guaranteed or endorsed by the publisher.
